# Comparison of long-term survival and immediate postoperative liver function after laparoscopic and open distal gastrectomy for early gastric cancer patients with liver cirrhosis

**DOI:** 10.1007/s10120-016-0675-4

**Published:** 2016-12-10

**Authors:** Amer Saeed Alshahrani, Ghung-Sik Gong, Moon-Won Yoo

**Affiliations:** Division of Stomach Surgery, Department of Surgery, Asan Medical Center, University of Ulsan College of Medicine, 88 Olympic-Ro 43-Gil, Songpa-gu, 05505 Seoul, Korea

**Keywords:** Gastric cancer, Laparoscopic gastrectomy, Liver cirrhosis, Survival

## Abstract

**Background:**

Several studies have suggested no difference in the liver function of early gastric cancer (EGC) patients with liver cirrhosis (LC) between laparoscopic and open distal gastrectomy. However, the number of patients and comparison of long-term survival rates between the two groups are limited. The purpose of this study was to compare the long-term survival and immediate postoperative liver function of EGC patients with LC after laparoscopic and open distal gastrectomy.

**Materials and methods:**

The clinical data of EGC patients with LC who had no other malignancy and underwent distal gastrectomy at Asan Medical Center between January 2005 and April 2013 were investigated retrospectively. All patients were divided into two groups: the open group (OG) and laparoscopic group (LG). The clinicopathologic data of the two groups were compared.

**Results:**

The number of patients in each group was 48 and 27 in the OG and LG, respectively. There were no significant differences in the age, sex ratio, ASA score, cause of liver cirrhosis, preoperative Child-Pugh classification, tumor location, TNM stage, total postoperative drain amount, albumin, total bilirubin, alkaline phosphatase, alanine aminotransferase, prothrombin time, morbidity and recurrence rate. Shorter hospital stay, longer operative time and more retrieved lymph nodes were observed in LG. The long-term overall survival rate was not different between the two groups (*P* = 0.356).

**Conclusions:**

For EGC patients with liver cirrhosis, especially Child A cirrhosis, laparoscopic or laparoscopy-assisted distal gastrectomy can be a safe surgical procedure in comparison to open distal gastrectomy in terms of the long-term survival rate and immediate postoperative liver function.

## Introduction

Gastric cancer and liver cirrhosis are major public health concerns in Korea. Gastric cancer is the one of the most common cancers in Korea. The age-standardized incidence rate of gastric cancer is 59.3 and 23.5 per 100,000 people in males and females, respectively [1]. Chronic liver disease, including liver cirrhosis, is also very common in Korea, ranking as the eighth leading cause of death in South Korea in 2013. The mortality rate due to chronic liver disease in Korea was reported as 13.2 per 100,000 people with the peak mortality in middle-aged males, suggesting a heavy disease burden on families and society [2].

A study from Korea showed 2.0% of cirrhotic patients had gastric cancer. The study presented radical open gastrectomy with extended lymph node dissection as feasible in patients with compensated liver cirrhosis [3]. For patients with moderate to severe hepatic dysfunction, however, D1 or less extensive lymph node dissection with meticulous hemostasis may be the more reasonable surgical procedure, and radical gastrectomy is very dangerous, even fatal, for Child C patients [4, 5].

Although laparoscopic gastrectomy as the standard procedure for early gastric cancer is still a commentary on investigational treatment [6], the number of laparoscopic gastrectomies is increasing with comparable results to open gastrectomy [7], and laparoscopic gastrectomy can be considered an option in general clinical practice to treat cStage I cancer [8]. Although technically challenging because of portal hypertension, varices and thrombocytopenia, basic and advanced laparoscopic procedures may be safe for patients with Child A and B liver cirrhosis [9]. There are a few studies on laparoscopic gastrectomy for early gastric cancer in liver cirrhotic patients that have demonstrated that laparoscopic gastrectomy is a feasible surgical procedure in terms of postoperative liver function and complications [10, 11]. However, studies directed at the comparison of the long-term survival rate between open distal gastrectomy and laparoscopic distal gastrectomy are limited. In this study, we compared open distal gastrectomy and laparoscopic or laparoscopy-assisted distal gastrectomy for early gastric cancer in terms of immediate postoperative liver function and long-term survival in relation to oncology safety with more patients than previous studies.

## Methods

### Patients

Preoperative early gastric cancer (EGC) patients with liver cirrhosis who had no other malignant diseases, including hepatocellular carcinoma, and had undergone distal gastrectomy at Asan Medical Center between January 2005 and April 2013 were enrolled after institutional review board approval (#2015–1274).

### Preoperative evaluation

The patients were divided into two groups: open gastrectomy (OG) vs. laparoscopic or laparoscopy-assisted gastrectomy (LG). Liver cirrhosis (LC) was diagnosed based on the history of liver disease or a preoperative imaging study such as CT or USG. Age, sex, American Society of Anesthesiologists (ASA) score, the cause of liver cirrhosis, preoperative Child-Pugh classification, CEA/CA19-9/AFP, location of the tumor, TNM stage, daily and total postoperative surgical drain amount, pre- and postoperative albumin, total bilirubin, alkaline phosphatase, alanine aminotransferase and prothrombin time, postoperative morbidity, hospital stay after operation, the operative time, extent of lymph node dissection, count of retrieved lymph nodes, recurrence rate, recurrence-free survival and overall survival rates were analyzed.

### Surgical indications

Open or laparoscopic gastrectomy was chosen according to the preference and experience of the surgeons. In addition, the patients’ requests were considered in the choice of surgical procedure in the early period of this study because national medical insurance in Korea did not cover laparoscopic surgery and the costs of laparoscopic surgery were higher than those of open surgery.

### Surgical procedure

Laparoscopic gastrectomy was done with five ports and a 3-cm infraumbilical incision for telescope and specimen retrieval. The anastomosis was done intracorporeally with the Billroth type I, Billroth type II, Roux-en-Y gastrojejunostomy or uncut Roux-en-Y gastrojejunostomy reconstruction methods. Laparoscopy-assisted distal gastrectomy was done with five ports with a 4–5-cm upper midline incision for specimen retrieval and extracorporeal anastomosis. The pneumoperitoneum was maintained at 12 mmHg. LN dissection was scored according to the Japanese gastric cancer treatment guidelines [6]. To retract the liver, the attachment site of the lesser omentum to the right diaphragmatic crus was sutured intracorporeally, and then a thread (2/0 prolene straight^®^; Covidien, Mansfield, MA) was pulled by a suture passer was tied over the skin in the xiphoid process area [12].

### Data collection and statistical analysis

Electronic medical records, operative notes and pathological reports were retrospectively reviewed, and data were collected. SPSS version 20 software was used for statistical analysis. Chi-square test, Fischer’s exact test, Student’s *t* test and Kaplan-Meyer survival analysis with the log-rank (Mantel-Cox) test were used for comparison. *P* <0.05 was considered significant.

## Results

A total of 75 patients were enrolled in this study. The number of patients was 48 in the OG and and 27 in the LG. Patient demographics are summarized in Table 1. There were no significant differences in the age, sex ratio, underlying disease and preoperative Child-Pugh classification between the two groups. There were no Child C patients. The ASA score, cause of liver cirrhosis, location of the tumor, TNM stage, preoperative CEA/CA19-9/AFP, albumin, total bilirubin, alkaline phosphatase, alanine aminotransferase and prothrombin time all were not significant (Tables 2, 3). The operative time was longer and the numbers of retrieved lymph nodes were more in the LG than OG. The extent of LN dissection was significant. D0 occurred more in the OG and D2 more in the LG. Hospital stay was longer in the OG than LG (Table 3). During the first 6 days, postoperative liver function tests were not different, except for AST on the 3rd and 6th day after surgery, which was higher in the OG (Table 2).

**Table 1:** Patient demographics

Total no. = 75	OG (48)	LG (27)	*P* value
Age (years, mean ± SD)	58.4 ± 9.6	59.6 ± 9.7	0.607
Sex (male/female)	43/5	23/4	0.714
Underlying disease			
DM	14	3	0.090
HTN	13	11	0.303
Arrhythmia	1	0	1.00
Pulmonary TB	2	2	0.616
Previous abdominal surgery	0	1	0.360
ASA score			
1	2	0	0.561
2	41	24	
3	5	3	
Causes of cirrhosis			
HBV	29	11	0.044
HCV	5	0	
Alcoholic	7	9	
Unknown	7	7	
Child-Pugh class			
A	43	24	1.00
B	5	3	

*OG* open gastrectomy group, *LG* laparoscopic or laparoscopic-assisted gastrectomy group, *ASA* American Society of Anesthesiologists

**Table 2:** Pre- and postoperative day 1, 3 and 6 laboratory tests

Preop laboratory test	OG (48)	LG (27)	*P* value
Albumin (g/dl)	3.8 ± 0.6	3.7 ± 0.5	0.571
AST (IU/l)	45.5 ± 52.7	32.1 ± 13.8	0.100
ALT (IU/l)	34.7 ± 31.1	29.3 ± 18.4	0.408
Alkaline phosphatase (IU/l)	75.3 ± 23.9	90.7 ± 45.3	0.060
Total bilirubin (mg/dl)	0.9 ± 0.3	0.9 ± 0.3	0.852
Prothrombin time (INR)	1.1 ± 0.2	1.1 ± 0.1	0.603
Prothrombin time (%)	88.6 ± 17.6	89.1 ± 15.5	0.912
AFP (ng/ml)	21.9 ± 53.3	6.8 ± 9.0	0.437
CEA (ng/ml)	1.7 ± 1.2	2.2 ± 1.4	0.166
CA19-9 (U/ml)	17.9 ± 28.4	10.3 ± 9.3	0.260
POD#1 laboratory tests			
Albumin (g/dl)	3.0 ± 0.4	3.0 ± 0.3	0.821
AST (IU/l)	62.9 ± 77.0	61.0 ± 46.1	0.907
ALT (IU/l)	47.9 ± 61.4	54.9 ± 44.8	0.610
Alkaline phosphatase (IU/l)	59.0 ± 19.2	89.8 ± 117.6	0.189
Total bilirubin (mg/dl)	1.3 ± 0.5	1.3 ± 0.6	0.595
Prothrombin time (INR)	1.24 ± 0.3	1.26 ± 0.04	0.796
Drain amount (ml)	240 ± 305	220 ± 374	0.827
POD#3 laboratory tests			
Albumin (g/dl)	3.0 ± 0.4	2.9 ± 0.4	0.811
AST (IU/l)	39.3 ± 28.9	28.1 ± 10.5	0.024
ALT (IU/l)	38.4 ± 39.7	31.4 ± 21.6	0.407
Alkaline phosphatase (IU/l)	54.8 ± 14.4	58.1 ± 20.8	0.432
Total bilirubin (mg/dl)	1.3 ± 0.8	1.3 ± 0.7	0.726
Prothrombin time (INR)	1.25 ± 0.24	1.29 ± 0.03	0.613
Drain amount (ml)	341 ± 369	220 ± 338	0.200
POD#6 laboratory tests			
Albumin (g/dl)	3.1 ± 0.4	3.0 ± 0.3	0.641
AST (IU/l)	33.7 ± 12.6	28.5 ± 6.3	0.033
ALT (IU/l)	38.5 ± 34.5	22.6 ± 13.9	0.040
Alkaline phosphatase (IU/l)	65.4 ± 19.8	68.8 ± 29.8	0.606
Total bilirubin (mg/dl)	1.4 ± 1.5	1.2 ± 0.5	0.471
Prothrombin time (INR)	1.27 ± 0.28	1.23 ± 0.18	0.762
Drain amount (ml)	310 ± 352	173 ± 254	0.182

*OG* open gastrectomy group, *LG* laparoscopic or laparoscopic-assisted gastrectomy group, *POD#* postoperative day number

**Table 3:** Operative details

	OG (48)	LG (27)	*P* value
Extent of LN dissection^a^			
D0	17	2	0.002
D1	8	3	
D1+	14	6	
D2	9	16	
TNM stage			
Ia	44	25	0.741
Ib	3	2	
IIa	1	0	
Reconstruction			
GD	35	22	0.656
GJ	6	1	
GJJJ	5	3	
RYGJ	2	1	
Op time (min)	134.4 ± 41.0	153.4 ± 47.9	0.076
No. of retrieved LNs	20.3 ± 9.7	30.6 ± 15.3	0.003
Hospital stay (days)	14.5 ± 6.2	11.2 ± 3.7	0.005
Drain amount until POD#6	1851 ± 1772	1177 ± 1866	0.157

^a^According to the 2010 Japanese gastric cancer treatment guidelines (ver. 3) [6]

*OG* open gastrectomy group, *LG* laparoscopic or laparoscopic-assisted gastrectomy group, *LN* lymph node, *GD* gastrodeudenal anastomosis, *GJ* gastrojejunal anastomosis, *GJJJ* gastrojejunal with jejunojejunal anastomosis, *RYGJ* Roux-en-Y gastrojejunal anastomosis, *POD#* postoperative day number

### Morbidity and mortality

There was no significant difference in either the daily or total postoperative surgical drain amount, although the amount was less in the LG (1127 ± 1876 vs. 2037 ± 1763 ml in OG *P* value = 0.053). Nine patients (18.78%) had immediate postoperative complications in the OG; seven of them had grade 2 severity based on the Clavien-Dindo Classification, one patient had grade 1 and one patient had grade 5, who died from postoperative bleeding and liver failure in the OG. Four patients (14.8%) in the LG had immediate postoperative complications; two of them had grade 1 and two had grade 2, which were not statistically significant between the OG and LG (*P*  = 0.654). Details of complications are listed in Table 4.

**Table 4:** Morbidity and mortality

	OG (48)	LG (27)	*P* value
Complications severity^a^			
None	39 (81.3%)	23 (85.2%)	0.464
Grade I	1 (2.1%)	2 (7.4%)	
Grade II	7 (14.6%)	2 (7.4%)	
Grade V	1 (2.1%)	0 (0.0%)	
Morbidity			
None	39	23	0.514
Pneumonia	1	1	
Wound	1	2	
Leakage	1	0	
Bleeding	4	0	
Others	2	1	
Transfusion			
RBC	3	0	0.549
FFP	1	0	1.000
In-hospital mortality	1	0	1.000

*OG* open gastrectomy group, *LG* laparoscopic or laparoscopic-assisted gastrectomy group

^a^The Clavien-Dindo classification of surgical complications. Grade I: any deviation from the normal postoperative course without the need for pharmacological treatment or surgical, endoscopic or radiological interventions. Allowed therapeutic regimens are: drugs as antiemetics, antipyretics, analgesics, diuretics and electrolytes and physiotherapy, including wound infections opened at the bedside. Grade II: requiring pharmacological treatment with drugs other than those allowed for grade I complications. Blood transfusions and total parenteral nutrition are also included. Grade IIIa: requiring surgical, endoscopic or radiological intervention not under general anesthesia; grade IIIb: an intervention under general anesthesia. Grade IVa: a life-threatening complication (including CNS complications) + requiring IC/ICU management with single-organ dysfunction (including dialysis); grade IVb: multiorgan failure. Grade V: death of the patient

### Recurrence and long-term survival

Recurrences of gastric cancer were detected in three cases. First, a 62-year-old male with Child-Pugh A liver cirrhosis underwent open distal gastrectomy, gastrojejunostomy with Braun jejunojejunostomy and D0 lymph node dissection for EGC in the lesser curvature of the antrum. The pathologic stage was T1bN0M0. He was discharged on the 12th postoperative day without complications. Lymph node recurrence was detected in the lesser curvature side of the remnant stomach and supraceliac area 10.7 months after surgery. He died from gastric cancer progression 21.2 months after surgery. Second, a 48-year-old male with Child-Pugh A liver cirrhosis underwent open distal gastrectomy, gastroduodenostomy and D0 lymph node dissection for EGC in the lesser curvature of the antrum. The pathologic stage was T1bN0M0. He was discharged on postoperative day 14 after ascites and pleural effusion management. The hepatic recurrence was detected 24.4 months after surgery. He died from liver failure 34.6 months after surgery. Third, a 71-year-old male with Child-Pugh A liver cirrhosis underwent laparoscopy-assisted distal gastrectomy, gastroduodenostomy and D2 lymph node dissection for EGC in the greater curvature of the body. The pathologic stage was T1bN1M0. He was discharged on postoperative day 7 without complications. The hepatic recurrence was detected 3.8 months after surgery. He had been followed up for 17.2 months postoperatively but was then lost to follow up.

**Fig. 1:**
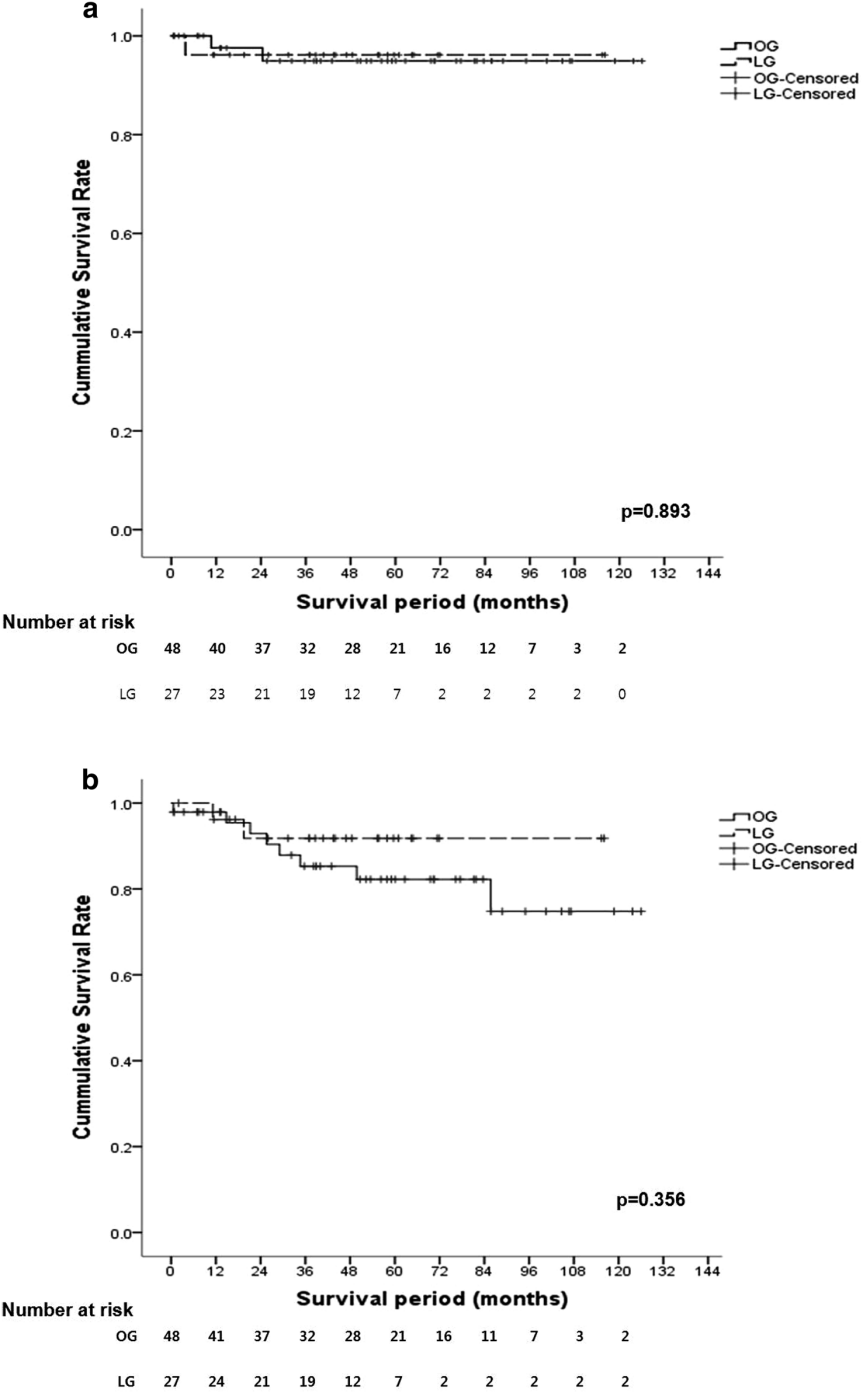
Long-term survival. **a** Recurrence-free survival; **b** overall survival

Regarding the operative procedure, the recurrence-free survival rate was also not different between the two groups (OG, 94.9%; LG, 96.2%; *P* = 0.893) (Fig. 1a). The overall survival rate was not different between the two groups (OG, 74.8%, LG, 91.8%; *P* = 0.356) (Fig. 1b). There was no significant difference in recurrence rate between the two groups (Table 5). In the long-term follow-up, eight patients died in the OG: two patients from gastric cancer, four patients from liver disease and two patients from other reasons, while two patients in the LG died from causes unrelated to gastric cancer (Table 5).

**Table 5:** Long-term recurrence and death

	OG (48)	LG (27)	*P* value
Mean follow-up period (month)	55.5 ± 35.9	46.8 ± 27.8	0.248
Recurrence	2	1	1.000
Cause of death			0.313
Gastric cancer	2	0	
Liver	4	1	
Other cause	2	1	

*OG* open gastrectomy group, *LG* laparoscopic or laparoscopic-assisted gastrectomy group

The overall survival rate according to the extent of lymph node dissection was compared, and no difference was observed (*P* = 0.394).

## Discussion

In a place with high incidence of gastric cancer and liver cirrhosis, surgeons often operate on gastric cancer patients with liver cirrhosis. Because of screening programs, early diagnosis, advanced surgical techniques and improving postoperative management, the mortality and morbidity associated with gastric cancer surgery have decreased at present.

Laparoscopic gastrectomy has become a common procedure for EGC in countries with a high incidence of early gastric cancer. Pneumoperitoneum and lymph node dissection were a concern for mortality and morbidity in laparoscopic gastrectomy for gastric cancer patients with liver cirrhosis because decreased portal venous return may lead to liver ischemia and increase ascites, and dissected lymphatic channels may increase portal pressure in case of cirrhosis [13].

In our study, during the first 6 days, postoperative liver function tests were not different, except for the AST on the 3rd and 6th day and ALT on the 6th day after surgery, which was higher in the OG than LG. Because AST and ALT were normalized from the 6th day after surgery in both groups, clinical impact was thought to be minimal. Its result was similar to that of other studies [11].

The total drain amount until the 6th postoperative day was not statistically different between two groups although the extent of lymph node dissection was less in the OG. This result did not support the concern of increasing ascites with more LN dissection. We think this is due to less visceral exposure and bowel manipulation in the LG in which more lymph node dissections were performed.

In terms of liver function and ascites, the morbidity in the LG was comparable to that in the OG. This showed the safety and benefit of the laparoscopic approach in liver cirrhosis patients.

The main disadvantage of LG is the longer operative time. There were no bleeding complications in the LG, which could be because of the better magnified visualization, use of ultrasonic shears for better hemostasis and the easy accessibility of the deep operative field. Cheng et al. reported a meta-analysis on the advantage of using ultrasonic shears compared conventional techniques: shorter operating time, reduced intraoperative blood loss and decreased drainage amount [14].

There is no guideline for lymph node dissection for gastric cancer patients with liver cirrhosis. The most common postoperative complication of these patients is massive ascites related to lymph node dissection [3, 15]. In this study, D2 lymph node dissection was more commonly performed in the LG although D2 lymph node dissection was not thought to be advisable for EGC patients even with liver cirrhosis. However, according to the Japanese guideline for cT1 N+ patients, standard gastrectomy with D2 lymph node dissection is recommended. Under laparoscopic view, the size of the lymph nodes might be exaggerated compared to the real ones, and operators might regard these lymph nodes as metastatic ones and perform D2 lymph node dissection during laparoscopic surgery. Similar results could also be found in other Korean studies in which D2 lymph node dissection for gastric cancer patients with liver cirrhosis was performed in 9 out of 18 (50%) during laparoscopy-assisted distal gastrectomy [11].

According to liver function, the cause of death can differ. The causes of death for gastric cancer patients with Child A cirrhosis and Child B or C were usually related to gastric cancer recurrence and liver failure/hepatocellular carcinoma, respectively [4, 16]. Therefore, D2 lymph node dissection can be recommended for gastric cancer patients with Child A cirrhosis while less than D2 lymph node dissection can be considered for gastric cancer patients with Child B or C. Furthermore, hepatocellular carcinoma can develop in early gastric cancer patients with liver cirrhosis after gastric cancer surgery. Ikeda et al. reported the most common cause of death in EGC was hepatocellular carcinoma (4 of 9 deaths) [17]. Therefore, during the postoperative follow-up period, early diagnosis of hepatocellular carcinoma is important for EGC patients with liver cirrhosis.

The decision concerning the treatment strategy is more complicated in case of gastric cancer patients with Child B rather than Child A cirrhosis. Both the life expectancy of patients with liver cirrhosis and the chance of gastric cancer progression should be considered in the treatment strategy. However, there have been few studies about the indications for gastrectomy for gastric cancer patients with Child B cirrhosis. Lee et al. just showed the overall 5-year survival rate of gastric cancer patients (51 stage I patients out of a total 94 patients) with Child A and B and C cirrhosis was 69.1 and 44.4%, respectively [3]. Considering this result, gastrectomy can be recommended even for patients with Child B cirrhosis, with the overall 5-year survival rate being over 40% in Child B and C if gastric cancer was detected at an early stage. Before surgery, downstaging of Child B to Child A cirrhosis is required if possible.

Our study has certain limitations. First, this study was retrospective and just carried out at a single center. Therefore, it had the limitation of generalization. Furthermore, the allocation of the operative approach to either LG or OG included a selection bias. The OG group might have included more patients with poor physical condition than the LDG group, although the ASA score and Child-Pugh classification showed no significant difference between the two groups. Second, an important factor during surgery is the amount of intraoperative blood loss, especially in liver cirrhosis patients. In this study, however, the data were not available for all the patients and could not be collected precisely. Therefore, the transfusion amount could be compared between the two groups indirectly, and no difference was observed. Third, there was no information about ligation of the hepatic branch from the left gastric artery in each group. These could be really important data on the immediate liver function and long-term outcomes of liver cirrhosis patients. However, data about aberrant hepatic branch ligation could not be used in this retrospective study. Similarly, portal hypertension also can affect postoperative outcomes. However, accurate data about portal hypertension could not be collected because of the limitations of retrospective data.

## Conclusion

For EGC patients with liver cirrhosis, especially Child A cirrhosis, laparoscopic or laparoscopy-assisted distal gastrectomy can be a safe surgical procedure for comparison of open distal gastrectomy in terms of the long-term survival rate and immediate postoperative liver function.
